# The Design and Material Characterization of Reclaimed Asphalt Pavement Enriched Concrete for Construction Purposes

**DOI:** 10.3390/ma13214986

**Published:** 2020-11-05

**Authors:** Jaroslav Pokorný, Radek Ševčík, Jiří Šál

**Affiliations:** 1 Department of Civil Engineering, Faculty of Technology, Institute of Technology and Business, Okružní 517/10, 370 01 České Budějovice, Czech Republic; jaroslav.pokorny@mail.vstecb.cz (J.P.); sal@mail.vstecb.cz (J.Š.); 2 Institute of Theoretical and Applied Mechanics of the Czech Academy of Sciences, Prosecká 809/76, 190 00 Praha, Czech Republic

**Keywords:** concrete, reclaimed asphalt pavement, construction industry

## Abstract

Reclaimed asphalt pavement (RAP) is a valuable commodity originating during processes of road/highways rehabilitations, resurfacing in the cases of the revelation of underneath-placed layers. Removed material can be successfully recycled and utilized as a supplementing material for new hot asphalt mixes. However, its dosages are limited because of variations in properties of aged bitumen compared to fresh material and, thus, a significant amount of waste material is remaining as waste products. Nonetheless, this commodity may find usage in the construction industry that suffers from a shortage of high-quality and easily available aggregates. This work aimed to investigate the optimal substitution of mined natural aggregate with commonly available RAP in order to produce composites with the comparable mechanical performance of reference ordinary Portland concrete. The aggregate substitutions up to 100% with RAP have been studied with a combination of mechanical and analytical techniques. Obtained experimental data showed changes in the porous structure, mineralogy, and in the amount of formed cement-related hydration products that influenced the mechanical performance of produced composites. Composite with 10% of natural aggregate substitution with RAP has shown the strength class C16/20 after 28 days of water curing, according to the EN 206-1. Such innovative products could be utilized in the construction industry. The usage of waste RAP could contribute to preservation of our environment for future generations.

## 1. Introduction

With regard to the development of society and urbanism, concrete-based composites and all modifications constitute a fundamental and available building material all over the world. The main advantages of concrete structures are considered to be quick construction processes, high compressive strengths (even in term of a few days), durability, etc. [1]. In this context and according to the available data, the world concrete production covers about 25 billion tons annually and many predictions claimed that its consumption will have a growing tendency in the future [2,3]. Commonly used concrete consists of three fundamental constituents: Portland cement, blend of fine and coarse aggregates, and batch water. The aggregate mix fills ~75% of the volume unit of produced concrete. Therefore, the quality of used aggregates and their overall interactions with cement-based matrix are crucial for the quality of formed concrete [4].

Currently, many natural sources of high-quality aggregates are depleted or their stocks are significantly decreasing, which is the cause of their increasing costs on the market [5]. The global aggregate consumption for the construction industry exceeded 48.3 million tons annually [6]. Another factor affecting final aggregate cost is related to energy consumption for quarrying and subsequent manufacturing. In addition, costs related to transportation of raw materials for long distance have to be considered as well [7,8]. Different waste materials were tested to partially and/or full replacement of natural aggregates [9,10]. At the present time, the commonly used coal-derived aggregates start to became a scarce commodity on the market and, thus, the usage of different waste materials is intensively investigated and their advantages/disadvantages were reported e.g., in Reference [11] and references therein.

Reclaimed asphalt pavement (RAP), which is one of the waste materials, is obtained as a removed product of road/highways rehabilitations, resurfacing in the cases of the revelation of underneath-placed layers [12]. The recycling procedure of asphalt pavement is known and applied in more than one century. Nevertheless, the main extend of RAP recycle started during the oil crisis in 1973 [13,14]. Asphalts are usually composed of granular aggregate (often high-quality limestone) and bitumen in the percentage representation of 95% and 5%, respectively [15]. The mentioned material is applied for road structures in the form of Hot Asphalt Mixes (HAM) that can occur after the end of their service life recycled [16,17]. However, obtained RAP in the portion not exceeding 25% for new mix design is recommended only [16,18]. If more than 25% of RAP is used, higher stiffness of aged asphalt leads to structure cracking even at low temperatures and a higher inclination to fatigue compared to fresh raw materials [19]. Additionally, about 85.1 million tons of RAP was stockpiled in the U.S. in 2015 [8,20] and, in comparison, about 50 million tons of RAP is produced annually in Europe [13,14]. Notwithstanding, only limited research papers focused on the investigation of RAP as a potential candidate for natural aggregates replacement can be found [15,21,22]. Singh et al. [21] investigated the usage of adjusted RAP by the Abrasion and Attrition method in concrete mixes with w/b = 0.38, thus natural aggregate was substituted by RAP in the range of 25–100 wt.%. Reported results showed the decrease in strength with increasing RAP content. However, the application of treated RAP with lower asphalt content caused the decrease of mechanical resistivity by only about 20% even if 100% of natural aggregates were replaced with RAP. In addition, it was shown that the presence of RAP enhanced the workability of fresh composites, thus allowing long distance transportation. Al-Mufti and Fried [15] applied RAP as a replacing material of 20-mm gravel in concrete with a water to binder ratio of 0.5 and it was shown that 25% replacement decreased compressive strength by 27% of 28 days water-cured samples. Coppola et al. [22] investigated RAP (in the fraction up to 20 mm) substitution of 20% of coarse silica aggregate with ambiguous results. Obtained results showed a beneficial effect of RAP on workability, but, on the other hand, the mechanical performance of composites with 15% of RAP were found to be two times lower in the comparison with reference samples. Mahmoud et al. [23] applied the RAP aggregate as a substitution of a natural aggregate in the range from 0% up to 50% in self-consolation concrete mixes. The gradual decrease of slump flow values of fresh concrete mixtures together with a decrease of strength performance with increasing RAP incorporation were reported. Dosages up to 25% were determined to be suitable in order to preserve sufficient strength properties. Huang et al. [24] studied the influence of fine RAP (0.0/4.0 mm) and coarse RAP (4.0/20.0 mm) as full replacement of natural aggregate. Results showed systematic reduction of compressive strength reaching up to 27.6% in the comparison with the reference sample.

The main aim of this paper refers to the clarification of the role of RAP on properties of concrete composites and to design RAP-containing concrete composites of class C 16/20 suitable for construction purposes. Composites with different RAP/natural aggregates ratios were produced following standardized procedures and the obtained specimens were tested for their structural properties and mechanical resistance. Furthermore, the influence of RAP on the hydration reaction and morphology of concretes was studied with a combination of instrumental methods, such as X-ray powder diffraction and scanning electron microscope.

## 2. Experimental Part

### 2.1. Used Materials

**Figure 1:**
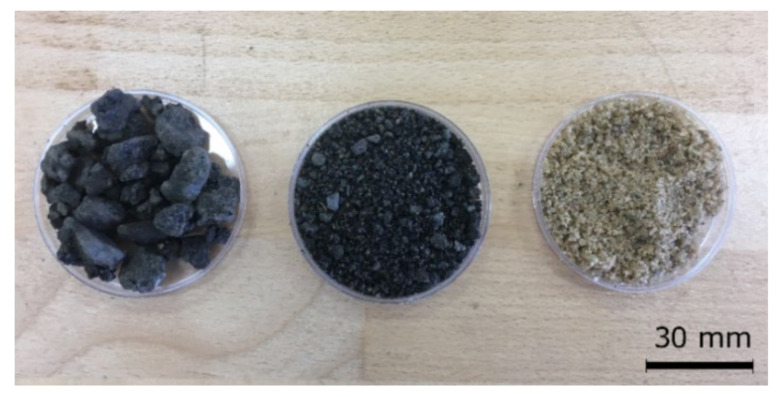
Samples of silica aggregate and RAP aggregate after separation of coarse particles.

**Figure 2:**
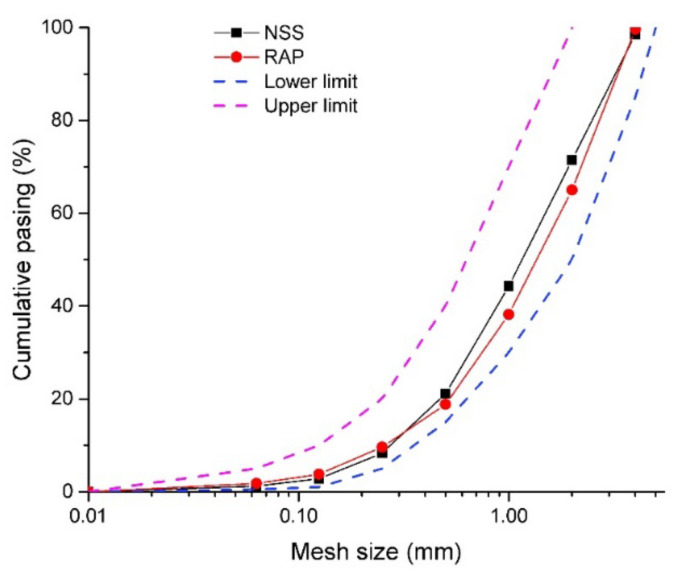
Particle size distribution of silica sand and RAP.

Ordinary CEM II/B-M 32.5 R (CEM II) conforming to the EN 197-1 [25] for experimental investigation was used. The binder comprised an average alite (C_3_S) content of 7.13 wt.% and possessed a relatively high specific surface area exceeding 380 m^2^·kg^-1^. Chemical composition and basic material characteristics, reported by the producer (Cemex, Ltd., Prachovice, Czech Republic), are listed in Table 1 and Table 2. Two sorts of fine aggregates in the fraction of 0/4 mm were used in this study. Mined Nature Silica Sand (NSS) was delivered by Mane Holding, Plc., České Budějovice, Czech Republic, and meets requirements for concrete manufacturing according to EN 12 620 [26]. This silica aggregate was substituted by Reclaimed Asphalt Pavement (RAP) obtained as a recycled material when resurfacing the road I/3 close to Benešov town, Czech Republic. The surface layer of asphalt pavement was removed by a cold milling machine and stockpiled in the nearby landfills. Collected RAP aggregate comprised a mix of coarse particles up to 20 mm and fine fractions below 4 mm. In order to get a comparable particle size distribution (PSD) with NSS, fine aggregates were separated by sieving with a 4-mm mesh sieve. The comparison of RAP before and after sieving together with NSS is depicted in Figure 1. The comparison of PSD of aggregates used in this study is reported in Figure 2. Lower and upper limits highlighted in PSD curves specify an optimal area in which granulometry of aggregate is suitable for concrete manufacturing according to EN 206-1 [27] and EN 12 620 [26].

### 2.2. Mix Design and Preparation of Test Specimens

The reference mix (RC) was designed according to the T.C. Kennedy method [33]. This method takes into consideration the quality of applied aggregate in dependence on the shape of grains, the rock type, and its technological processing. Reference mix was comprised of 362.7 kg of CEM II, 1 760.0 kg of NSS, and 199.5 kg of water for 1 m^3^ of produced fresh concrete. Based on the control mix, three types of RAP fine-grained concrete mortars were derived (see in Table 3), whereas natural silica sand (NSS) was substituted with RAP in the amount of 10 (RA-C 10), 50 (RA-C 50), and 100 vol.% (RA-C 100). Both aggregate types were dried before application. The amount of batch water was kept constant for all prepared mixtures with a w/c ratio of 0.55.

Input raw materials were blended with the mixer Spar 200 with vertical axes allowing simulate mixing conditions according to the EN 196-3 [31]. First, dry components including Portland cement, silica aggregate, or a blend of silica aggregate and RAP were homogenized for 60 s at 94 rpm. Then, batch water was gradually added for 30 s and mixing continued 30 s further. Then, sediments of dry material at the bottom of mixing vessels were removed and new mixing at 198 rpm for 120 s was applied. Tested specimens according to EN 12390-2 [34] were prepared. Fresh concrete was placed into iron molds, confirming the requirements of the EN 12390-1 [35], in the shape of prisms (100 × 100 × 400 mm), cylinders (diameter of 100 mm, the height of 200 mm), and cubes (edge of 100 mm). Each specimen was cast in one step and subsequently compacted with the help of a vibrating table (Matest, S.P.A., Treviolo, Italy) operating with the frequency 40 Hz (2400 oscillations per minute) for 2 min. Filled molds were covered by plastic foil and left in laboratory conditions at a temperature of 20 ± 1 °C and 45 ± 5% of relative humidity. After 24 h, partially hardened specimens were demolded and placed in a water environment at a temperature of 20 ± 1 °C for 27 days. The photographic gallery of prepared specimens is depicted in Figure S1 in Supplementary Materials.

### 2.3. Instrumental Techniques

At first, the material properties of the input components were analyzed. The specific gravity with the pycmometric method according to EN 1097-7 [29] was measured whereas the expanded combined uncertainty was about 1.6%. The powder density of CEM II and loose bulk density of both aggregates were obtained on the bases of the standard EN 1097-3 [28]. Compacted bulk density was determined with the same procedure. Only in the case of aggregate samples, the frequency of vibrating table was set to 40 Hz. In both cases, the expanded combined uncertainty reached 2.3%. Voids percentage with the expanded combined uncertainty of 4.4% was subsequently calculated from known values of loose bulk density and specific gravity of a particular aggregate type. PSD of aggregates was measured with the usage of sieving analysis, according to the standard EN 933-1 [36]. Tested samples were dried in hot air dryer at 105 ± 5 °C until the constant mass was reached. After subsequent cooling, representative aggregate samples were sieved through the set of sieves having a mesh size of 4.0, 2.0, 1.0, 0.5, 0.25, 0.125, and 0.063 mm and shaken by vibratory apparatus Retsch AS 200 (Germany). Water absorption of both NSS and RAP was measured in conformity with EN 1097-6 [37]. 

The consistency of fresh mixes was controlled with a slump test (EN 12350-2 [38]) performed at the end of mixing. The bulk density values were measured according to EN 12390-7 [39] whereas the expanded combined uncertainty of the applied method was 2.5%. Specific densities of hardened tested specimens were measured using the apparatus AccuPyc II 1340 (Micrometrics Co., Ltd., Norcross, USA). The relative standard deviation of specific density was determined to be lower than 1.1%. Total open porosity was calculated from obtained bulk and specific density values. The expanded combined uncertainty of total porosity was 5%. Flexural and compressive strength tests were performed with the usage of hydraulic press Servo Plus Evolution (Matest, S.P.A., Treviolo, Italy) with a disposing loading capacity of 3000/600 kN. Prisms with dimensions of 100 × 100 × 400 mm in a three-point bending setting were tested. On cubes (100 × 100 × 100 mm) and cylinders (diameter of 100 mm, height of 200 mm) maximum compressive forces were recorded and subsequently converted to compressive strengths. The expanded combined uncertainty of compressive strength was 1.8% and 2.0% in the sense of flexural strength determination.

The Mercury Intrusion Porosimetry (MIP) method was employed to determine the specimen’s porosity. The set of Pascal devices, Pascal 140 and Pascal 440, producing external pressure of 140 kPa and 440 MPa, respectively, were used. In general, siliceous samples are usually dried at 105 °C in order to remove excess moisture [40]. However, such high temperatures may cause the leakage of asphalt binder in pores in cement-based matrix and, thereby, the analysis can be adversely affected [41]. It was, therefore, approached to vacuum oven drying at 50 ± 5 °C until the constant sample mass was reached.

The adhesion of used aggregates with cement matrix was observed with a polarizing microscope equipped with a digital camera Olympus E-600. Pieces with area of 15 × 15 mm and thickness of 10 mm were cut from composites specimens. After rough grinding, samples were placed into plastic cylindrical molds and casted by epoxide resin Araldite 2020 under vacuum. After hardening at 21 ± 1 °C and 45 ± 5% of relative humidity in order to achieve a smooth surface, samples were ground with an automatic grinder equipped by SiC papers of a grain size of P 800, P 1200, P 2500, and P 4000. After that, samples were hand-polished with a diamond suspension of particle size of 1, 0.25, and 0.1 µm. Prepared polished sections were washed with 96% ethanol (p.a., Lach-Ner, Neratovice, Czech Republic).

The mineralogical composition of hydrated cement pastes was analyzed with X-ray powder diffraction (XRPD) employing a diffractometer D8 Advance (Bruker, Karlsruhe, Germany) with Bragg-Brantano geometry and equipped with LynxEye detector using CuKα radiation and Ni filter. Data were recorded in the angular range from 15 to 90° (2θ) with a step of 0.01° and counting time 0.4 s. Rietveld refinement method was used for a quantitative phase analysis in Topas 4.2 software (Bruker).

Prepared samples were observed under scanning electron microscope (SEM) Quanta 450 FEG (FEI, Brno, Czech Republic) using a backscatter electron detector. Prior to observations, samples were goal-coated (thickness of 5 nm). The acceleration voltage was set to be 20 kV.

## 3. Results and Discussion

### 3.1. Material Properties and Morphology of Used Aggregates

The material properties of NSS and its replacing RAP material are summarized in Table 4. In the case of RAP, lower loose bulk density and specific density by more than 20% and 8%, respectively, were detected. This finding pointed out the lower unit weight of produced composites with RAP aggregate regard to traditionally incorporated silica sand. Such light-weighted composites will reduce transportation costs. On the other hand, approximately two times higher water absorption of RAP in comparison with NSS was observed. Such a significance increase in water absorption is assumed to angular-shaped grains of RAP with the rough surface [42].

**Figure 3:**
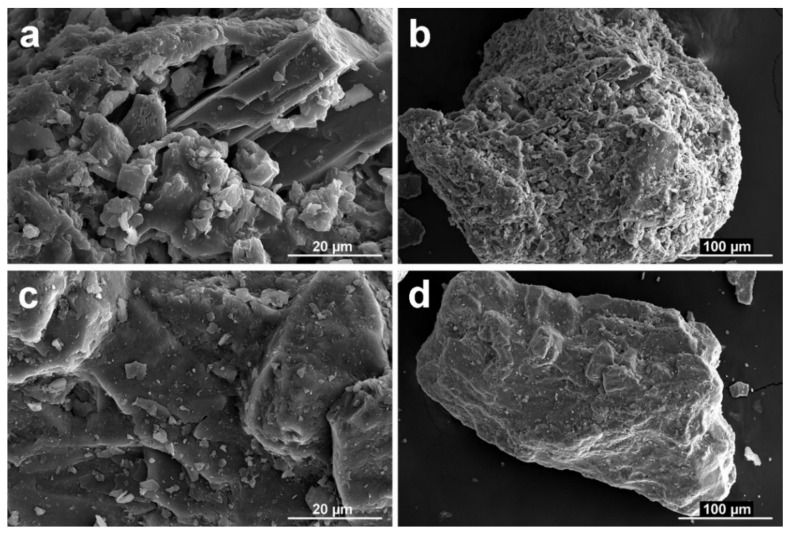
SEM image of RAP (**a**,**b**) and NSS (**c**,**d**) sample.

SEM images of NSS and RAP aggregates are depicted in Figure 3. Micrographs of RAP shows fine aggregate particles fully incorporated within the asphalt matrix (Figure 3a,b). Moreover, the presence of micro filler and agglomerated dust particles on the surface of bulky grains was observed (Figure 3b). These adsorbed predominantly flat-shaped particles with apparent highly specific surface area can be, to a certain extent, responsible for increased water absorption of asphalt-covered aggregate as already reported in Reference [43]. In addition, bonding ability of dust and agglomerated particles with coarser RAP grains can be importantly limited and, thus, in a certain way, it may be responsible for the limited strength properties of produced composites [21]. In contrast, silica sand grains represent a much smoother surface with randomly occurring particles of a few micrometers in size. This surface morphology of natural silica sand particles seems to be beneficial in term of uniform redistribution of batch water that is subsequently available for hydration of cement grains.

### 3.2. Characterization of Fresh Mixtures

Physical properties determined for fresh concrete mixes are given in Table 5. The addition of waste aggregate resulted in a visible workability deterioration of developed mixes. In this context, performed slump tests revealed the decrease of the initial slump with respect to the control mix of about 29.4% and 70.6% for the concrete mix with 50% and 100% contents of RAP, respectively. The control mix with a measured slump value of 170 mm met the class S4, according to the EN 206-1 [27]. This phenomenon can be connected, on the one hand, to the angular shape of asphalt-covered aggregate and, on the other hand, to its considerable water absorption (two times higher as discussed above) in comparison with NSS. Similar findings were reported in the study performed by Singh et al. [42]. Accordingly, with higher RAP incorporations into the concrete mix, the amount of water needed to preserve required workability should be increased or a plasticizer should be added. In addition, an adverse effect of bleeding caused by high dosages of RAP exceeding 50 vol.% was detected after several minutes once the specimens were casted. To avoid the bleeding issue, Coppola et al. [22] recommended to limit the amount of reclaimed asphalt to 15 wt.%. However, replacing of NSS with RAP helped to mitigate the unit weight of fresh concretes up to 12 wt.% in comparison with the reference mix. Although lower dosages of RAP (below 10%) did not reduce the weight of fresh mixes with respect to RC, the slump mitigation recorded was only 6% [22].

### 3.3. Structural Properties

Structural properties, bulk density, specific density, and total porosity of investigated samples are given in Table 6. The substitution of natural sand by artificial aggregate led to a decrease in bulk densities by 1.1% recorded for concrete with 10 vol.% of RAP, 4.4% determined for concrete with 50 vol.% of RAP, and 9.9% in the case of silica sand-free samples. In a similar way, specific density values of modified samples were reduced. Due to the gradual decline of both bulk and specific densities, total porosity values showed a growing trend. The total porosity of control specimens reached 17.6% contrary to 21.6% observed for material without the presence of natural silica sand. On the contrary, the difference in porosity of RC and RA-C 10 samples were found to be negligible. The incorporation of reclaimed asphalt pavement into concrete mixes partially decreased their unit weight, which introduces a beneficial aspect in view of dead load reduction in relation to bearing structures.

### 3.4. Mechanical Resistance

**Figure 4:**
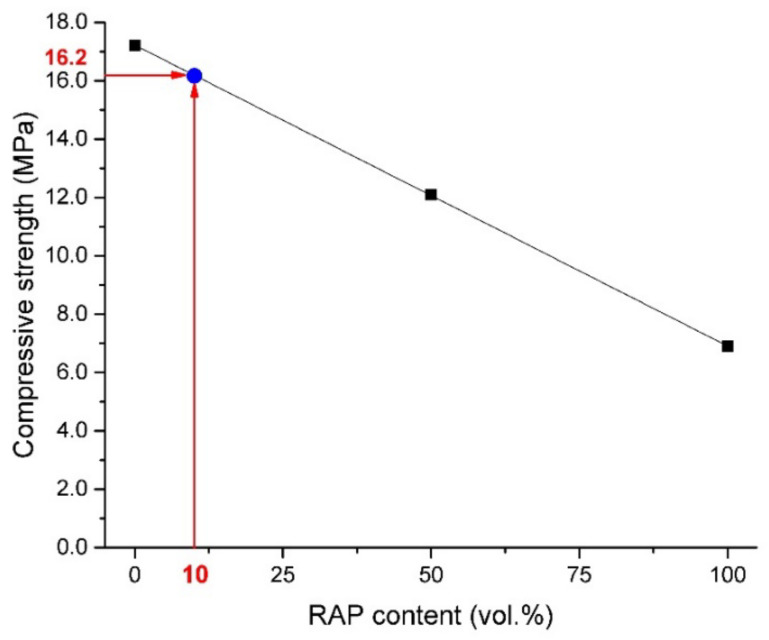
Comparison of compressive strengths obtained on cylindrical samples of concretes with a different content of the RAP aggregate.

**Figure 5:**
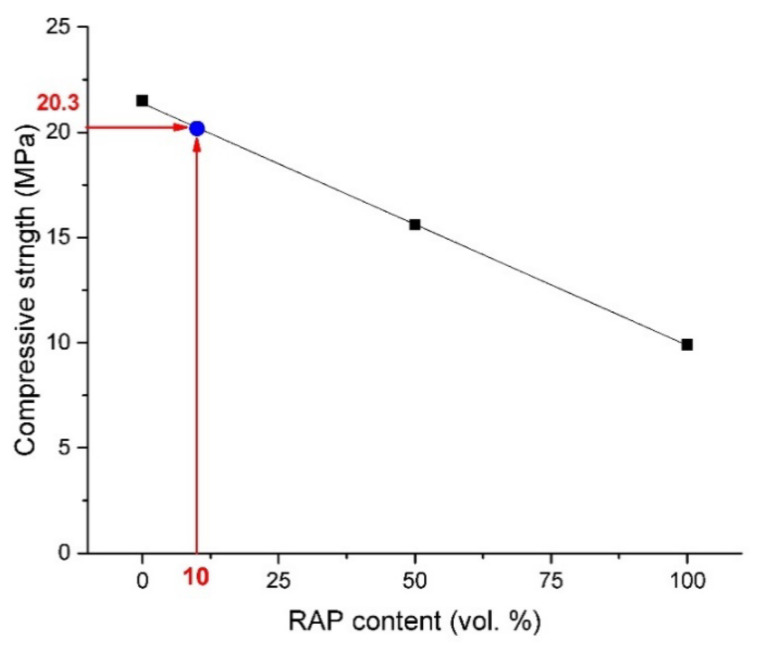
Comparison of compressive strengths obtained on cubic samples of concretes with a different content of the RAP aggregate.

**Figure 6:**
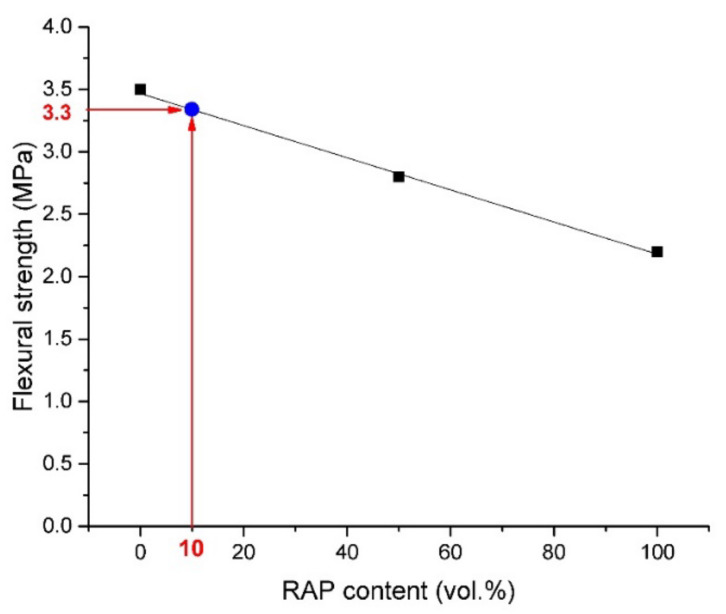
Flexural strength development of concretes with a different content of the RAP aggregate.

Flexural and compressive strength results obtained for all prepared fine-grained concretes, expressed as average values from six independent measurements, are shown in Figure 4, Figure 5 and Figure 6. Linearly decreasing trends with regression coefficient ~0.999, ~0.999, and 0.997 determined for compressive strengths measured on cylinders, cubes, and for flexural strength measurements, respectively, were plotted. This negative effect on the mechanical performance could be ascribed, in particular, to oil traces that may influence the hydration process of cement [22] and to weak bonds of the interfacial transition zone (ITZ) between cement paste and bitumen covered aggregate [44,45]. The compressive strength obtained on cylinders decreased from the initial value of 17.2 MPa detected for RC to 11.1 MPa (RA-C 50) and 6.9 MPa (RA-C 100). It represents the decrease of 35.5% and 59.9% with respect to reference samples. A similar trend was observed for cubic samples. The compressive strength was found to decrease of 35.4% for RA-C 50 and 54.0% for RA-C 100 in the comparison to the RC sample. The flexural strength was detected to decrease by 20.0% and 38.3% for RA-C 50 and RA-C 100, respectively, in the comparison with reference.

According to trends plotted in Figure 4, Figure 5 and Figure 6, RAP content of 10 vol.% seems to be optimal to produce composites of the strength class C 16/20. Such a composite should dispose of the average compressive strength of 16.2 MPa measured on cylinders and 20.3 MPa measured on cubes. In the case of flexural performance, RAP content limited to 10 vol.% should result in the strength of 3.3 MPa. Based on this prediction, a composite with only 10 vol.% of RAP (called RA-C 10) was produced by following the same procedure and being tested after 28 days of curing. The experimental values of compressive and flexural strengths were detected to be slightly higher than expected theoretical strengths. The compressive strength was found to be 16.6 MPa and 21.1 MPa measured on cylinders and cubes, respectively. The flexural strength was detected similarly to the theoretical prediction—3.3 MPa.

### 3.5. Mercury Intrusion Porosimetry (MIP)

**Figure 7:**
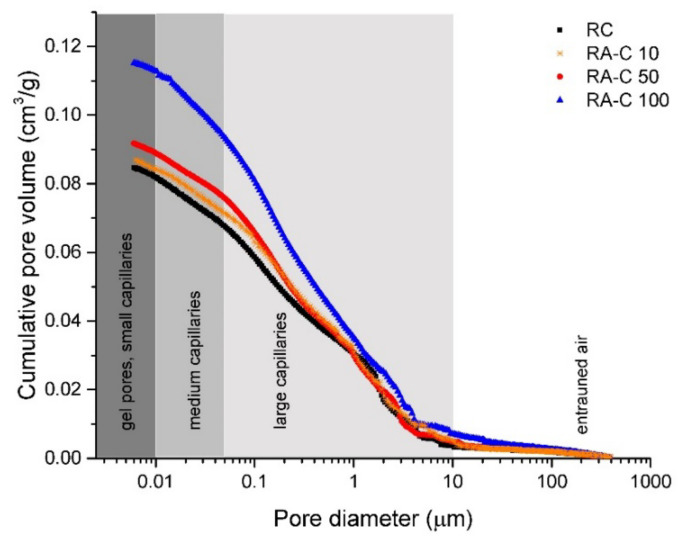
Cumulative pore volume curves obtained for reference (RC) and RAP (RA-C 10, RA-C 50, RA-C 100) specimens.

**Figure 8:**
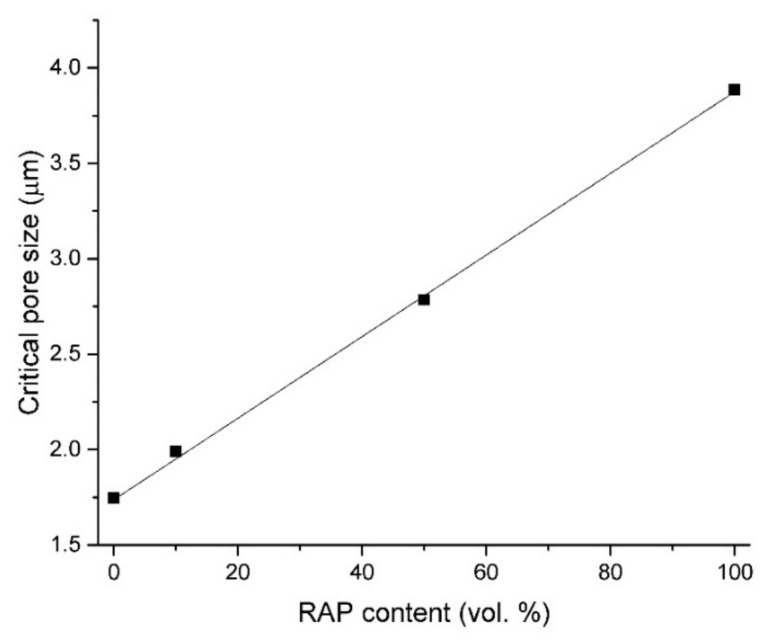
Critical pore diameter (*d*_cr_) in dependence on RAP content in concrete mixes.

**Figure 9:**
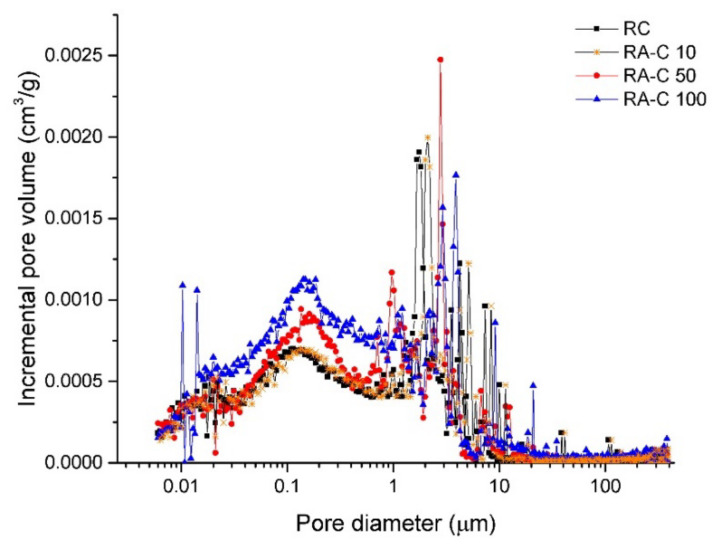
Incremental pore volume curves obtained for reference (RC) and RAP modifies (RA-C 10, RA-C 50, RA-C 100) specimens.

Cumulative pore volume cures measured on tested concretes are given in Figure 7. The gradual increase of intruded volume accompanied with increased NSS substitutions with RAP was found to be in agreement with the structural properties mentioned above (and summarized in Table 5) and also with the observed decreasing mechanical resistivity of tested samples. In Figure 8, the dependence of the critical pore size (*d*_cr_) on RAP content in studied mixtures is depictured. A critical pore size refers to the steepest slope of the cumulative pore curve and its value is derived from differential distribution curves shown in Figure 9. The procedure for obtaining this parameter is described in detail in Reference [46]. The *d*_cr_ characterize the interconnection of pores that allow more efficient water transport and, thus, the higher rate of transmissivity for chemical substances through the cement matrix [46]. Plotted values of *d*_cr_ in the dependence for increasing RAP content in concrete showed a growing linear trend with a high regression coefficient of ~0.999. It indicates a large volume of interconnected pores in RAP samples increasing with the higher substitutions of NSS.

The pores sizes classification, graphically highlighted in Figure 7, may be used as a tool revealing important changes in porous structures of tested concretes caused by RAP additions. Pores could be sorted according to their size into several groups: gel pores and small capillaries (diameter of 0.0025–0.01 µm), medium capillaries (diameter of 0.01–0.05 µm), large capillaries (0.05–10 µm), and entrained air (diameter higher than 10 µm) [47,48,49]. The main changes were detected in pores with a diameter below 1 µm and above 3 µm. The group of entrained air is composed of spherically shaped pores and helps to decrease the risk of damage by physically and chemically related volumetric changes (frost, salts) [46]. Gel and capillary pores play an essential role on transport processes and permeability in cement-based materials [50]. The RA-C 50 sample showed only a slightly difference in the porous structure, particularly in the area of gel pores and capillaries, in comparison with reference material. In view of concrete without silica sand, the porosity increase is more apparent in both the capillary/gel pores region and in the entrained air section. On the contrary, 10 vol.% of NSS substitution with RAP had a minimum impact on the porosity growth, which indicates the cumulative pore volume curve obtained for concrete RA-C 10.

### 3.6. Mineralogical Composition

Mineralogical compositions of the RAP aggregate as well as developed reference and RAP-enriched concretes are listed in Table 7. RAP, as an input raw material, contained predominantly mineral phases, such as quartz, calcite, albite, and orthoclase. Looking at reference concrete composition (RC), quartz in the amount exceeding 50 wt.% was recorded due to usage of silica aggregate in concrete mix. However, with the growing RAP dosage, quartz content was detected to gradually decrease up to 16.8 wt.% (RA-C 100). The wt.% of minerals, such as muscovite, clinochlore, amphibole, and orthoclase presented in raw RAP increased gradually with the higher content of RAP in produced composites. The same tendency was found for the amount of an amorphous phase. Some changes in Portland cement-related hydration products formation were observed as well, especially the increased formation of ettringite in the case of composites containing RAP. The portlandite formation rate increased around 1 wt.% for both RA-C 50 and RA-C 100 concretes in comparison to a reference sample. Sample RA-C 10 showed only a minor increase (about 0.2 wt.%) in Portlandite content with respect to the reference. It was reported that the formation of Portlandite could be affected by possible leaching of organic compounds from RAP in pore solution [51]. The content of calcite and albite increased gradually with the increasing RAP dosages, which suggest the increment of cementitious products [52].

### 3.7. Microscopic Analysis

**Figure 10:**
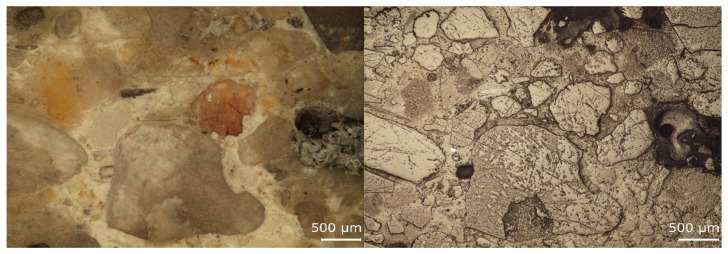
Microscopic images of the RC concrete sample observed under unpolarized (**left**) and polarized (**right**) light.

**Figure 11:**
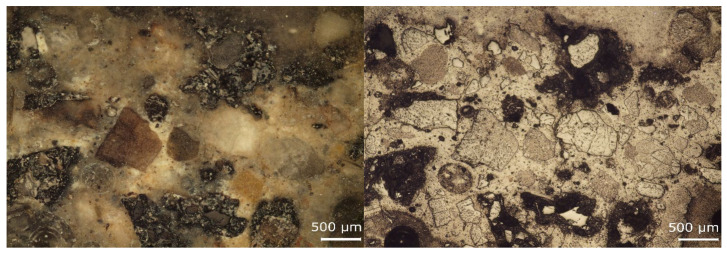
Microscopic images of concrete sample RA-C 50 observed under unpolarized (**left**) and polarized (**right**) light.

The quality of Interfacial Transition Zone (ITZ) was initially evaluated on prepared polished sections. The images of reference material (RC) and concrete with 50 vol.% of RAP are depicted in Figure 10 and Figure 11. The sample with NSS contentment showed very compact adhesion and interaction of aggregate grains and cement matrix. There is no evidence of porous layers and damages of the ITZ in the surrounding of coarser particles. In the case of samples with RAP, it is visible with the naked eye that aggregate grains are mostly covered with aged bitumen. Additionally, the preferential adhesion of cement matrix was with asphalt layers. The more detailed observations were performed using SEM.

**Figure 12:**
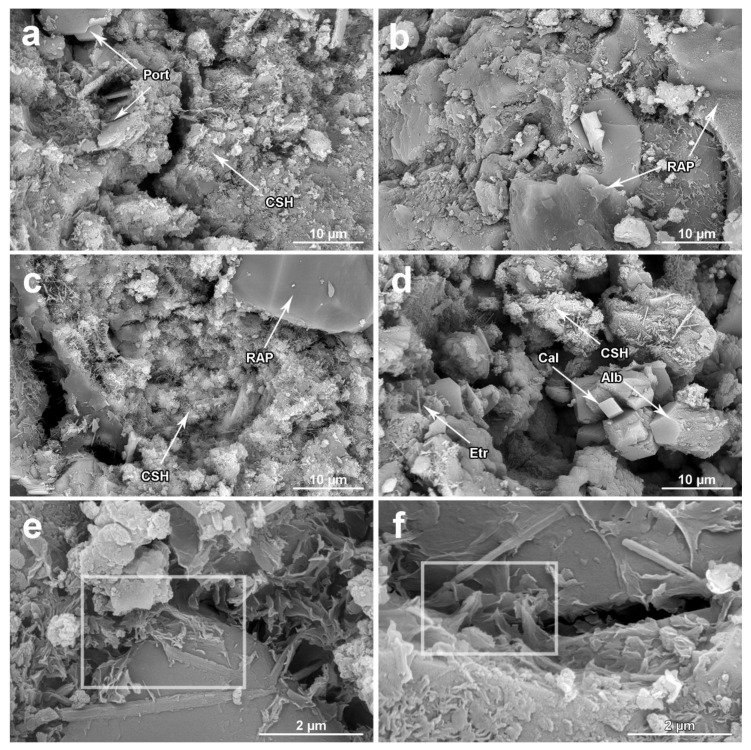
SEM micrographs of RC (**a**), RA-C 10 (**b**), RA-C 50 (**c**), RA-C 100 (**d**) specimens and an examples of ITZ zones (highlighted within inserted rectangles between RAP and cement matrix (**e**) and in the system of RAP-cement-RAP) (**f**). Some phases were identified according to their typical morphology.

Images of the microstructure of all tested composites including the reference sample after 28 days of water curing are shown in Figure 12. Some phases, based on their morphology, were indicated (Port—Portlandite, CSH—calcium silicate hydrate, Etr—ettringite, Cal—calcite, Alb—albite). The inner structure of hydrated RC consists of well observed, larger crystals of Portlandite (Port) surrounded by hydrated calcium silicate (CSH) and gels creating the main solid skeleton. In addition, smaller irregular-shaped crystals of vaterite supplement the overall composition. Focusing on the RAP modified sample, all the above-described mineral and gel phases were observed as well. The higher abundance of calcite (Cal) and albite (Alb) in samples with RAP confirmed the results of quantitative phase analysis of obtained XRPD patterns. If the samples without and with RAP are compared, it is possible to notice larger voids and a less compact microstructure in composite structures, especially for RA-C 50 (Figure 12c) and RA-C 100 (Figure 12d). Such observations correspond to the measured higher values of open porosity and reduced mechanical resistance as discussed in the previous text. The ITZ between RAP and cement matrix and the bonding of RAP grains with cement are depicted in Figure 12e,f. Only limited adhesion of cement to RAP is visible in both cases. Consequently, the asphalt film act as a separating layer hindering the creation of hard and durable cement-related bonds on the aggregate surface, which was already reported in Reference [53]. These observations were found to be in line with the observed samples after mechanical testing. Crack propagations during compressive strength tests were mainly noticed close to RAP grains rather than at the interfacial of silica aggregate. The cement matrix was recorded.

With respect to experimental analyses performed, reclaimed asphalt pavement introduces a valuable replacing commodity to natural silica sand in the sense of the preparation of composite materials intended for the building industry. However, RAP dosages in concrete mixes are limited because of its specific material properties that differ from those measured for dense aggregates types commonly used in the concrete industry.

## 4. Conclusions

The potential usage of reclaimed asphalt pavement as a substitution of mined nature silica aggregate for the design of concrete C 16/20 for construction purposes was studied. Properties of raw input materials as well as developed concretes with various RAP content after 28 days of water curing were examined and completed by a combination of experimental procedures and instrumental techniques. Obtained data showed notable effects of natural silica sand substitutions with waste RAP aggregate on the behavior of the ordinary Portland concrete as follows.

- The behavior of fresh mixes was affected by specific properties of RAP, in particular, importantly increased water absorption, compared with NSS, which resulted in the considerable mitigation of workability. The slump test revealed very good workability of reference mix (slump of 170 mm) meeting the class S4 while the full replacement of NSS with RAP resulted in a very stiff mixture inappropriate for construction purposes. Moreover, high dosages of RAP reaching up 100% led to the bleeding of placed concretes. Reduced RAP dosages up to 10 vol.%, however, mitigated the value of slump in about 6% only, thereby the class S4 was kept.
- Reclaimed asphalt pavement addition helped to decrease the unit weight of produced concretes, thereby the transportation costs can be partially reduced. However, a lowered weight of RAP-dosed concretes is accompanied with the increase in total porosity by 0.6% (50% of RAP content) and 3.6% (100% of RAP content) with regard to the control samples. At lower NSS substitutions with a tested alternative material, not exceeding 10 vol.%, this adverse effect will be negligible. Increased critical pore size induced by waste aggregate brought the growth of interconnected pores and the increase of medium-sized capillaries responsible for transport processes in porous structure. On the contrary, detected higher presence of entrained air, in the form of isolated bubbles, in RAP-enriched concretes can result in improved frost and salt resistivity.
- Incorporation of the waste aggregate resulted in the deterioration of the strength resistance of developed concretes, linearly proportional to its dosage. With the total substitution of NSS with RAP, the average compressive strength measured on cylinders decreased by 60% and by 54% in the case of cubic specimens when compared to the reference material. The maximum flexural strength drop reached approximately 38%. From interpolation of obtained experimental data, the concrete with RAP content of 10 vol.% was found to be optimal in the sense of meeting requirements for the class C 16/20. The experimental mechanical testing of prepared samples with 10 vol.% of reclaimed asphalt (RA-C 10) revealed the average compressive strength of 16.6 MPa and 21.1 MPa for cylindrical and cubic samples, respectively. The flexural strength was determined to be 3.3 MPa. Thus, the design concrete with 10 vol.% substitution of NSS with RAP met requirements for practical usage in civil engineering applications.
- The mineralogical composition obtained by XRPD analysis showed the increased contents of calcite, albite, muscovite, and amorphous phase induced with the increased substitutions of NSS with RAP. On the other hand, silica was detected to gradually decrease. Moreover, the ettringite was detected to be higher in samples containing RAP. SEM observations shown the morphology of hydration products and/or phases are presented in RAP. Microscopic analysis revealed prevailing adhesions occurring between asphalt cover presented on aggregate grains and cement matrix, which cause the decreased mechanical performance of concretes with high RAP content.

From the provided experimental investigation, it can be deduced that reclaimed asphalt pavement can be potentially applied in the concrete C 16/20 production as a partial substitution of nature silica aggregate in limited amount up to 10 vol.% in order to maintain strength properties. The incorporation of unutilized waste aggregate help save depleted nature aggregate recourses and mitigate quarrying-induced impacts on the environment. The findings obtained in the field of the pore structure of produced composites showed the increased potential of RAP-dosed concretes in terms of improved durability that could be the aim of future research.

## Figures and Tables

**Table 1:** Chemical composition and basic material properties of used cement.

Oxide Composition (wt.%)	
SiO_2_	15.84
Al_2_O_3_	4.57
Fe_2_O_3_	2.33
CaO	62.69
MgO	2.64
K_2_O	0.63
Na_2_O	0.25
SO_3_	2.93
Cl^-^	0.08

**Table 2:** Material properties of CEM II.

Characteristic	CEM II	Standard
Powder density (kg·m^−3^)	965	EN 1097-3 [28]
Specific density (kg·m^−3^)	3090	EN 1097-7 [29]
Specific surface area (m^2^·kg^-1^)	344	EN 196-6 [30]
Initial setting time (min)	181	EN 196-3 [31]
Final setting time (min)	273	
Loss on ignition (wt.%)	4.55	EN 196-2 [32]

**Table 3:** Composition of produced concretes.

Material	Content (kg·m^−3^)			
	RC	RA-C 10	RA-C 50	RA-C 100
CEM II	362.7	362.7	362.7	362.7
NSS 0/4 mm	1760.0	1584.0	880.0	-
RAP 0/4 mm	-	148.1	740.7	1481.4
Water	199.5	199.5	199.5	199.5

**Table 4:** Physical properties of used aggregate types.

Characteristic	NSS	RAP
Loose bulk density (kg·m^−3^)	1592	1340
Compacted bulk density (kg·m^−3^)	1810	1610
Specific density (kg·m^−3^)	2517	2310
Water absortion (%)	0.78	1.55
Voids (%)	37	42

**Table 5:** Properties of fresh concrete mixes.

Property	RAP Content (Vol.%)			
	0	10	50	100
Initial slump (mm)	170	160	120	50
Density (kg·m^−3^)	2322	2258	2183	2044

**Table 6:** Structural properties determined on dried hardened samples.

Property	RAP Content (Vol.%)			
	0	10	50	100
Bulk density (kg·m^−3^)	2078	2055	1987	1872
Specific density (kg·m^−3^)	2521	2498	2430	2387
Total porosity (%)	17.6	17.7	18.2	21.6

**Table 7:** Mix proportions of RC and RAP derived concretes.

Mineral Substance (wt.%)	RAP	RC	RA-C 10	RA-C 50	RA-C 100
Quartz—SiO_2_	20.0	50.8	46.3	29.1	16.8
Vaterite—CaCO_3_	-	4.3	4.2	3.5	4.6
Calcite—CaCO_3_	19.2	2.0	3.5	8.3	11.8
Alite—3CaO·SiO_2_	-	-	-	-	0.3
Belite—2CaO·SiO_2_	-	-	1.0	1.2	0.5
Portlandite—Ca(OH)_2_	0.1	1.4	1.6	2.2	2.1
Ettringite—Ca_6_Al_2_(SO_4_)_3_(OH)_12_·26H_2_O	-	0.8	1.8	4.3	3.0
Albite—NaAlSi_3_O_8_	23.1	6.5	8.9	14.4	15.1
Microcline—KAlSi_3_O_8_	0.6	6.7	6.7	7.2	2.8
Muscovite—KAl_2_(AlSi_3_O_10_)(F,OH)_2_	6.1	1.2	1.7	3.1	4.4
Clinochlore—(Mg,Fe)_3_(Si,Al) _4_O_10_(OH)_2_·(Mg,Fe)_3_(OH)_6_	6.1	-	1.0	3.3	1.1
Amphibole—(Mg,Fe)_4_Al(Si_7_Al)O_22_(OH,F)_2_	3.7	-	0.2	0.5	4.5
Orthoclase—KAlSi_3_O_8_	12.1	-	0.4	1.5	0.8
Amorphous content	9.0	16.4	17.3	21.4	32.2

## Supplementary Materials

The following are available online at https://www.mdpi.com/1996-1944/13/21/4986/s1, Figure S1: The collection of prepared prismatic concrete samples with dimensions of 100 × 100 × 400 mm of after flexural strength measurements: (a) RC, (b) RA-C 10, (c) RA-C 50, (d) RA-C 100.
