# Supplementary material for: The Design and Material Characterization of Reclaimed Asphalt Pavement Enriched Concrete for Construction Purposes
Source: Materials (Basel). 2020 Nov 5;13(21):4986. doi: 10.3390/ma13214986 (PMC7663929; doi:10.3390/ma13214986)
Supplement: Supplementary file 1 [file materials-13-04986-s001.pdf]

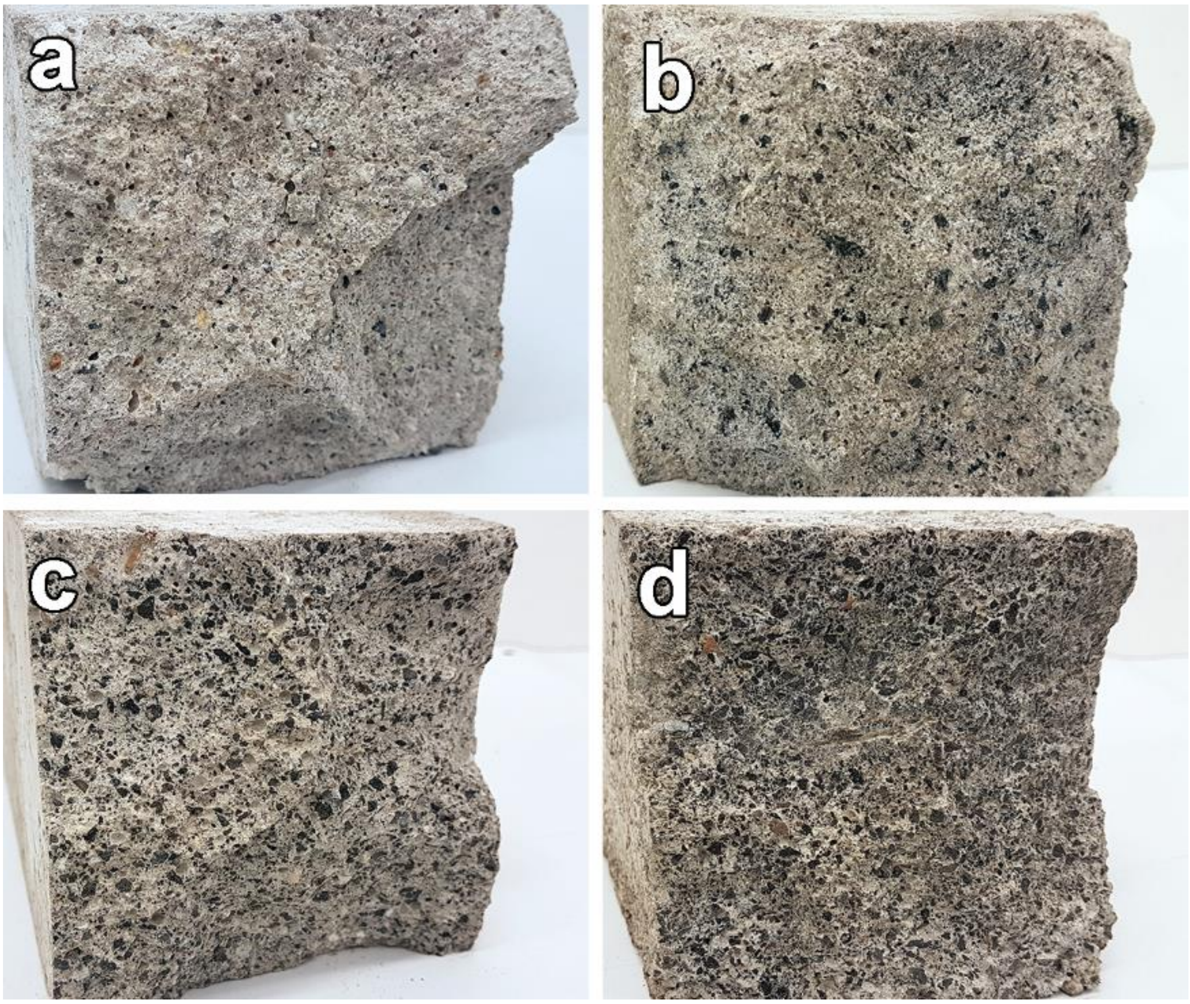

Fig. S1: The collection of prepared prismatic concrete samples with dimensions of 100 x 100 x 400 mm of after flexural strength measurements: a – RC, b – RA-C 10, c – RA-C 50, d – RA-C 100.
